# Short-Term Effect of a Wild Blueberry (*Vaccinium angustifolium*) Drink on Vascular Reactivity and Circulating Adhesion Molecules in Healthy Adults: A Double-Blind, Randomized, Placebo-Controlled, Crossover Trial

**DOI:** 10.3390/nu18142376

**Published:** 2026-07-21

**Authors:** Marco Rendine, Samuele Venturi, Mirko Marino, Daniela Martini, Patrizia Riso, Alberto Battezzati, Dorothy Klimis-Zacas, Cristian Del Bo’

**Affiliations:** 1 Department of Food, Environmental and Nutritional Sciences (DeFENS), Division of Human Nutrition, Università degli Studi di Milano, 20133 Milan, Italy; marco.rendine@unimi.it (M.R.); samuele.venturi@unimi.it (S.V.); mirko.marino@unimi.it (M.M.); daniela.martini@unimi.it (D.M.); patrizia.riso@unimi.it (P.R.); alberto.battezzati@unimi.it (A.B.); 2 International Center for the Assessment of Nutritional Status and the Development of Dietary Intervention Strategies (ICANS-DIS), DeFENS, Università degli Studi di Milano, 20133 Milan, Italy; 3 School of Food and Agriculture, University of Maine, Orono, ME 04469, USA; dorothea@maine.edu

**Keywords:** wild blueberry, randomized controlled trial, (poly)phenols, vascular function, reactive hyperemia index, arterial stiffness, E-selectin, CD-15, VCAM-1, humans

## Abstract

**Background:** Wild blueberries (WB), abundant in (poly)phenols such as anthocyanins and chlorogenic acids, have shown vascular benefits in several preclinical and epidemiological settings. However, evidence from human trials remains limited. This double-blind, randomized, placebo-controlled, crossover trial investigated the effects of the consumption of a WB drink on vascular reactivity and circulating adhesion molecules in healthy young adults. **Methods:** Participants received 300 mL of a WB drink (30 g of freeze-dried WB powder containing 740 mg total (poly)phenols, 460 mg total anthocyanins and 170 mg chlorogenic acid) or the same amount of a placebo (PL) drink. Blood pressure, heart rate, microvascular reactivity (i.e., reactive hyperemia index, RHI, lnRHI and FRHI) and arterial stiffness (i.e., augmentation index, AIx) were assessed at baseline and 2 h after consumption of the WB and PL drinks using Endo-PAT2000. In addition, plasma circulating adhesion molecules including vascular cell adhesion molecule-1 (VCAM-1), E-selectin, and cluster of differentiation 15 (CD 15 or Lewis x) were analyzed using ELISA. **Results:** Overall, analysis of variance did not show a significant effect of treatment on the modulation of markers of vascular function and adhesion molecules but revealed an effect of time, with an increase in terms of microvascular reactivity (RHI, *p* < 0.01; lnRHI, *p* < 0.05; FRHI, *p* < 0.001) and augmentation index (AIx, *p* < 0.01), and a reduction in the circulating levels of VCAM-1 (*p* < 0.001) following the intake of both WB and PL drinks. **Conclusions:** In conclusion, the intake of a WB drink failed to affect vascular function in young healthy subjects. Larger-scale and longer-term intervention studies, particularly in individuals at increased cardiovascular risk and in those with vascular dysfunction, are warranted to further explore the role of WB in modulating vascular reactivity.

## 1. Introduction

The endothelium, part of the tunica intima layer, plays a crucial role in the vascular system. It regulates the flow of nutrients and other substances, controls the fluid passage into tissues, and secretes vasoactive compounds, including vasodilator molecules such as nitric oxide and vasoconstrictors like endothelin-1 and other substances [1,2]. An imbalance between the vasorelaxant and vasoconstricting factors, favoring the latter, results in the disruption of this homeostatic system, leading to vascular dysfunction and promoting cardiovascular diseases (CVDs) [1,3,4]. Endothelial and vascular function is widely recognized as among the most critical indicators of future CVD events, the primary cause of death worldwide [5]. Vascular dysfunction is considered a key mediator in the development of atherosclerosis and may serve as an important predictor of future clinical outcomes. Atherosclerosis is a gradual, progressive condition that can begin in childhood, influenced by a combination of environmental factors and genetic predisposition [6,7]. Early atherosclerotic lesions appear as fatty streaks within the intimal layer of arteries during childhood [8]. This early onset suggests that endothelial dysfunction may also develop in younger individuals, a group often not studied. Lifestyle, in particular diet, is pivotal in the promotion of vascular health and the prevention of CVDs. In this regard, foods rich in bioactive compounds such as (poly)phenols (PPs) can potentially reduce the risk of cardiovascular diseases [9,10]. In fact, epidemiological studies have observed an inverse correlation between the consumption of PPs and both mortality and the risk for cardiovascular events [11,12].

Wild blueberry (*Vaccinium angustifolium*; WB) is a fruit particularly rich in PPs, including anthocyanins (ACNs), phenolic acids, proanthocyanidins, flavan-3-ols, and flavonols [13,14]. Additionally, WB contains fiber, vitamins and minerals that, together with PPs, may contribute to its beneficial effects on vascular function and CVDs [15,16,17]. Experimental evidence suggests that blueberry-derived PPs may improve endothelial function through multiple complementary mechanisms, including enhanced nitric oxide bioavailability, reduced oxidative stress, attenuation of vascular inflammation, and modulation of endothelial signaling pathways [15,18,19,20,21]. Furthermore, in vitro and animal studies suggest that WB and its PPs may play a significant role in modulating biomarkers linked to vascular function [11,18,19,20,21]. However, limited human intervention studies have evaluated the effect of WB in healthy young adults, with inconsistent results [22]. Previous studies carried out in our laboratories documented the potential health benefits of highbush blueberry on vascular function but mainly in smokers [23] and in subjects with vascular dysfunction [24], while in a previous study, we failed to document an effect in healthy individuals [25] probably due to the short length of time between the intake of blueberry and the measurement of peripheral arterial function (1 h after the intake) and/or the dose of PPs administered (e.g., 300 mg ACNs). This double-blind, randomized, placebo-controlled, crossover study aims to evaluate the effect of a portion of a WB (*Vaccinium angustifolium*) drink (300 mL), richer in PPs (approximately 740 mg of total PPs, 460 mg of ACNs and 170 mg of chlorogenic acid), on markers of vascular reactivity (i.e., reactive hyperaemia index; RHI) and arterial stiffness (i.e., augmentation index; AIx) in healthy young adults. Moreover, the levels of inflammation-related biomarkers, including the endothelial adhesion molecules vascular cell adhesion molecule 1 (VCAM-1) and E-selectin, together with cluster of differentiation 15 (CD15), a carbohydrate antigen involved in leukocyte adhesion, were also evaluated to support the findings and potentially explain the mechanisms of action.

## 2. Materials and Methods

### 2.1. Participant Recruitment

Twenty healthy young adults were recruited from the staff and student population of the University of Milan. Inclusion criteria were: (i) healthy subjects, (ii) age between 20–45 years, (iii) non-smokers, (iv) alcohol consumption limited to no more than 14 drinks of wine or beer per week. Women with regular menstrual cycles were included and evaluated during the same phase of the menstrual cycle for both intervention visits. Specifically, assessments were conducted during the late secretory phase, the week preceding menstruation, when estrogen and progesterone levels decrease, and follicle-stimulating hormone (FSH) and luteinizing hormone (LH) remain relatively low [26]. Exclusion criteria included: (i) obesity (BMI ≥ 30 kg/m^2^), (ii) hypertension (systolic blood pressure > 140 mmHg and/or diastolic blood pressure > 90 mmHg), (iii) history of cardiovascular diseases, diabetes, hepatic, renal or gastrointestinal diseases, (iv) allergy to blueberries. Furthermore, those who were following a specific diet (vegan or vegetarian) and those who had used medications, drugs, or supplements in the last month were excluded. Subjects’ eligibility was confirmed through a general anamnesis carried out by the medical staff of the International Center for the Assessment of Nutritional Status and the Development of Dietary Intervention Strategies (ICANS-DIS) of the University of Milan, through questions about health status and lifestyle, and a rigorous clinical evaluation. All participants signed the informed consent form before being enrolled. The study was in line with the Declaration of Helsinki and approved by the Ethics Committee of the University of Milan.

### 2.2. Wild Blueberry and Placebo Drink Preparation

The WB powder was supplied by Jasper Wayman & Sons (Milbridge, ME, USA) while the PL powder was provided by North Carolina Food Innovation Lab (Kannapolis, NC, USA). The nutritional composition of WB and PL powders has been determined in previous studies [27,28] and summarized in Table 1.

One portion (30 g) of freeze-dried WB and PL provided a similar amount and type of sugars and dietary fibre. Monomeric ACNs and chlorogenic acid in the WB accounted for 1.53% and 0.57% of the powder weight, respectively. Malvidin glycosides were the main ACNs in the WB powder (0.5%), followed by petunidin (0.27%), cyanidin (0.22%), and peonidin (0.1%) glycosides.

The WB beverage was prepared by mixing 30 g of freeze-dried wild blueberry (WB) powder, corresponding to approximately 200 g of fresh wild blueberries, with 300 mL of water to obtain a beverage that was as homogeneous as possible. The powder was mixed thoroughly in a dark glass bottle until fully dispersed. The placebo (PL) beverage was prepared following the same procedure and was formulated to match the sugar, fibre, and energy content of the WB beverage. It contained natural blueberry flavoring together with food-grade colorants (Purple Blend Lake, Red 40 Lake, Red 40 Dye, Blue 2 Lake, and Blue 2 Dye) to mimic the appearance and sensory characteristics of the WB beverage while containing no PPs. Additional information on the composition of both powders is available from the manufacturer (North Carolina Food Innovation Lab, Kannapolis, NC, USA). Both WB and PL beverages were prepared fresh each day and stored at 4 °C until consumption to preserve product stability. Participants were instructed to consume the beverage within 10 min to standardize the timing of consumption across all participants. As a small amount of insoluble material could remain because of the natural fibre content of both powders, participants were instructed to rinse the container with water and consume any residual material using a spoon to ensure complete ingestion of the product.

### 2.3. Experimental Design

**Figure 1:**
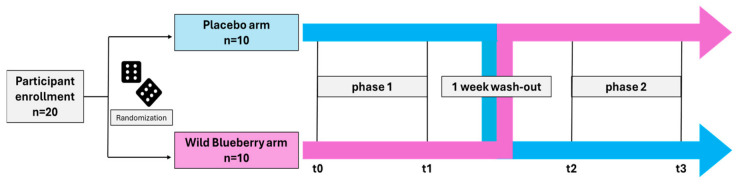
Schematic representation of the randomized, double-blind, crossover study design. Each participant completed two intervention days (Phase 1 and Phase 2) separated by a washout period of at least one week. On each intervention day, baseline measurements were taken in the morning under fasting conditions (t0 for Phase 1, t2 for Phase 2), followed by consumption of the WB or PL drink. Post-consumption measurements were performed 2 h later (t1 for Phase 1, t3 for Phase 2). The order of treatments was randomized.

The study was a randomized, double-blind, placebo-controlled, crossover trial. Participants were randomized to one of the two treatment sequences using a computer random number generator (www.randomizer.org). Both participants and investigators involved in beverage administration, outcome assessment, and statistical analyses remained blind to treatment allocation until completion of the study and data analysis. Participants were asked to consume a WB or PL drink on one intervention day, followed by a wash-out period of at least 1 week, after which the treatments were switched. Volunteers received dietary indications for the days before the interventions: these included avoiding a list of PP-rich foods such as berries, unpeeled apples, red/purple fruits and vegetables, red wine, and chocolate. Tea and coffee could be consumed up to three times per day. Additionally, subjects were asked to standardize their meals and to avoid physical activity during the days preceding each experimental session. Compliance with the dietary instruction was assessed by conducting a 24-h dietary recall for the day before each experiment. On each intervention day, peripheral arterial function and blood pressure were measured in the morning at baseline (t0 for phase 1, and t2 for phase 2) in fasting subjects and 2 h after the consumption of the WB or PL drink (t1 for phase 1, and t3 for phase 2). This timing corresponds to the two intervention periods illustrated in Figure 1. This protocol was chosen based on previous studies carried out in our laboratories showing an increase in plasma PPs and an improvement in peripheral arterial function 2 h after the consumption of blueberries [23,24,29,30].

### 2.4. Evaluation of Physical Activity Levels and Adherence to the Mediterranean Diet

The levels of physical activity were evaluated through the International Physical Activity Questionnaire (IPAQ), which estimates total energy expenditure [31], while the adherence to the Mediterranean diet (MeD) was evaluated by the MedScore and the MEDI-LITE questionnaires [32,33].

### 2.5. Anthropometric Measurements

Anthropometric measurements, including body weight and height, were obtained during the screening visit according to international guidelines [34]. Body mass index (BMI) was subsequently calculated as weight (kg) divided by height squared (m^2^).

### 2.6. Blood Pressure

Blood pressure was measured before and after peripheral arterial function measurement. The evaluation included both systolic (SBP) and diastolic blood pressure (DBP) measured in a seated resting position following the JNC 7 guidelines [35].

### 2.7. Peripheral Vascular Function and Arterial Stiffness

The peripheral vascular function was assessed as endothelial-dependent vasodilation in the small finger arteries using Endo-PAT2000 (Itamar Medical Ltd., Caesarea, Israel), a non-invasive plethysmographic method, as described in previous studies [23,36]. Briefly, pulsatile volume changes in the fingertip are detected by a pressure transducer located at the end of each probe. Subjects were instructed to maintain a supine position, with both hands at the same level, in a comfortable, dark, climate- and noise-controlled environment (22–24 °C), to minimize interexperimental variability. After a 10-min equilibration period, the blood pressure cuff on the study arm was inflated to 220 mmHg for 5 min to occlude the brachial artery. The cuff was then deflated to induce reactive hyperemia, and signals from the probes were recorded for 5 min by the software. At the end of the test, a reactive hyperemia index (RHI) was calculated as the ratio of the post-to-pre-occlusion peripheral arterial tonometry amplitude of the tested arm and was normalized to the corresponding signal in the contralateral control arm. According to the commonly adopted EndoPAT reference threshold, RHI values < 1.67 have been associated with impaired endothelial function and increased cardiovascular risk, although this cut-off has been primarily validated in middle-aged and at-risk populations [37]. Additionally, the EndoPAT-2000 provided other indices including the Framingham RHI (FRHI), calculated using a different post-occlusion hyperemia period (90–120 s) without baseline correction factor, the digital AIx, a measure of pulse wave reflection and a marker of arterial stiffness, which is derived from digital pulse volume waveforms and is determined from the baseline pulse wave shape, and finally the AIx adjusted to a heart rate of 75 bpm (AIx@75), as digital AIx is inversely and linearly influenced by heart rate [38]. It should be noted that this normalization is based on a fixed, population-averaged correction and does not necessarily eliminate the within-sample dependence of AIx@75 on heart rate, particularly in a small cohort with a relatively narrow heart-rate range.

### 2.8. Blood Sampling

Blood samples were collected at baseline and after 2 h from the consumption of WB or PL for the general biochemical assessment and the analysis of the different markers. Specifically, blood was collected after the vascular function evaluations to avoid potential side effects on vascular reactivity. Samples were drawn into tubes containing EDTA as an anticoagulant for plasma and silicone gel for serum. Plasma and serum were separated by centrifugation at 2300× *g* for 15 min at 4 °C, aliquoted and stored at −80 °C until analysis of markers of vascular health.

### 2.9. Analysis of Biochemical Parameters

A general biochemical assessment was performed, including glucose and insulin concentrations, lipid profile (triglycerides, TG; total cholesterol, TC; HDL-cholesterol, HDL-C; LDL-cholesterol, LDL-C), hepatic function (alanine aminotransferase (ALT), aspartate aminotransferase (AST), gamma-glutamyl transferase (GGT)), and high-sensitivity C-reactive protein. All these parameters were assessed at baseline, using standard laboratory analysis. LDL-C was calculated using the Friedewald method.

### 2.10. Evaluation of Markers Related to Vascular Health

Plasma samples collected before and after WB drink consumption were used to quantify markers related to vascular function and inflammation, including vascular cell adhesion molecule-1 (VCAM-1; Cat. No. 455623), E-selectin (Cat. No. MBS164784-96), and cluster of differentiation 15 (CD 15 or Lewis x; Cat. No. MBS7244209-96), using ELISA kits according to the manufacturer’s instructions (MyBioSource Inc., San Diego, CA, USA). Following incubation with antibodies and fluorophore, fluorescence was measured using a TECAN Infinite F200 plate reader. Plasma concentrations were calculated using a standard curve generated by a 4-parameter algorithm. A specific dilution factor was applied as needed.

### 2.11. Statistical Analysis

The sample size was determined based on the expected variation in RHI, as the primary endpoint of this trial. Based on our previous studies evaluating the acute effects of blueberry intake on vascular function [24,30], we estimated that 16 participants would provide 80% statistical power to detect a biologically relevant change in RHI (a difference in RHI of 0.30, with a standard deviation of the within-subject difference of 0.40) at an α level of 0.05. Since the present study adopted a crossover design, each participant received both interventions and served as his/her own control, thereby reducing inter-individual variability. To account for potential dropouts, 20 participants were recruited. The Statistical Package for Social Sciences (SPSS) v.29. (IBM Corp., Armonk, NY, USA) was used to conduct statistical analyses. The Shapiro–Wilk test was applied to verify the normal distribution of the variables. Pearson correlation analyses were conducted to determine the associations between MeD adherence, physical activity, and cardiovascular markers. To exclude any potential carry-over effect, an analysis of variance (ANOVA) was performed with the treatment sequence (WB drink versus PL drink or vice versa) as the independent factor. Subsequently, because no carryover effect was detected, the data were analyzed using repeated-measures ANOVA. This analysis included treatment (WB drink vs. PL drink) and time (before and after each treatment) as within-subject factors to assess the impact of the WB drink on the variables of interest. The difference in responses between the WB and PL drink periods was assessed based on statistically significant *p*-values for the interaction between time and treatment in the overall repeated measures ANOVA. Post hoc analysis between treatments was evaluated using the least significant difference (LSD) test. An exploratory subgroup analysis was performed in participants presenting baseline endothelial function values below the commonly used EndoPAT threshold (RHI < 1.67). This analysis was conducted to investigate whether the vascular response to the interventions differed among subjects with relatively lower endothelial function at baseline. Partial eta squared (η^2^p) values are reported as measures of effect size for the time × treatment interaction in the repeated-measures ANOVA analyses. Since blood pressure was measured both before and after the EndoPAT assessment for each intervention, the mean of the two measurements was used for statistical analyses.

For all statistical analyses, significance was set at *p*-value ≤ 0.05.

## 3. Results

### 3.1. Participant Enrollment and Baseline Characteristics

**Figure 2:**
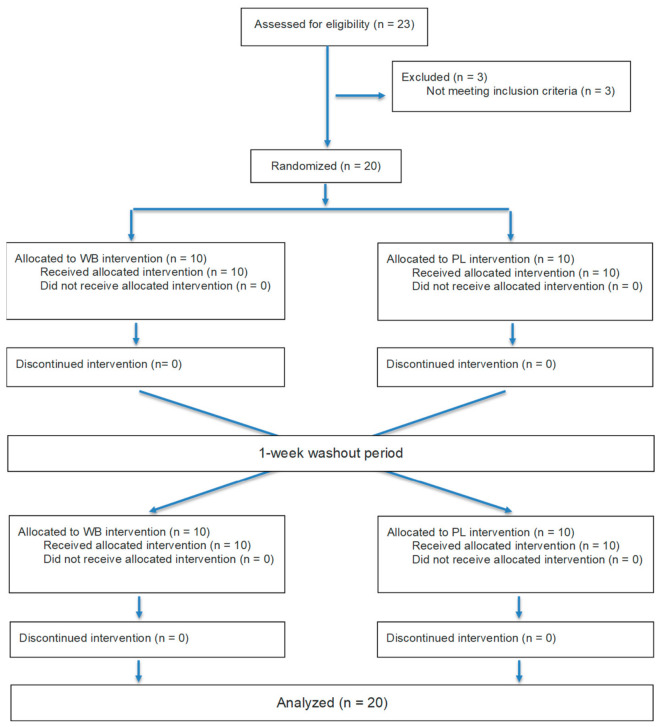
CONSORT flow diagram of participant progression through the randomized, double-blind, placebo-controlled crossover trial. Participants were randomly assigned to one of two treatment sequences (wild blueberry followed by placebo, or vice versa), separated by a washout period of at least one week. All randomized participants completed both intervention periods and were included in the final analysis. WB = wild blueberry; PL = placebo.

The participant flow throughout the study is summarized in the CONSORT flow diagram (Figure 2), prepared according to the CONSORT 2025 statement [39]. Briefly, 23 individuals were assessed for eligibility (of whom 20 met the inclusion criteria), were randomized, and completed both intervention periods.

The demographic and clinical characteristics of the subjects are reported in Table 2. A balanced cohort of 10 males and 10 females was included in the study. All the participants completed the trial. Subjects were healthy, with a mean age of 29.3 years and a mean BMI of 22.2 kg/m^2^. Notably, 12 out of the 20 subjects (60%) exhibited vascular dysfunction (RHI < 1.67), while the mean AIx was negative (−12.4%) for most of the subjects, indicating a low grade of vascular stiffness.

### 3.2. Pearson Correlation Analyses of Markers at Baseline

**Figure 3:**
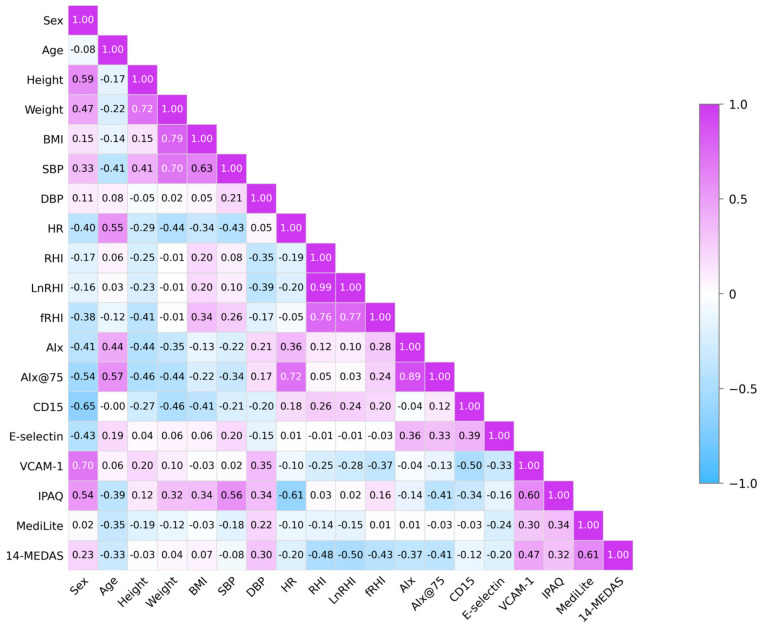
Correlation matrix between adherence to the Mediterranean Diet, MET and markers of vascular health. AIx = augmentation index; AIx@75 = augmentation index normalized to a heart rate of 75 bpm; CD15 = cluster of differentiation 15; DBP = diastolic blood pressure; FRHI = Framingham reactive hyperemia index; E-selectin = endothelial selectin; HR = heart rate; IPAQ = International Physical Activity Questionnaire; PL = placebo; RHI = reactive hyperemia index; SBP = systolic blood pressure; VCAM-1 = vascular cell adhesion protein-1. Colors indicate the strength and direction of correlations: darker shades represent stronger correlations, while lighter shades represent weaker correlations. Positive correlations are shown in purple, and negative correlations in blue.

Figure 3 presents the Pearson correlation matrix of markers at baseline. Among the most relevant correlations, sex (0 = female, 1 = male) was negatively associated with AIx@75 (r = −0.535, *p* = 0.01) and CD15 levels (r = −0.615, *p* = 0.001), while positively correlated to VCAM-1 levels (r = 0.696, *p* < 0.0001) and METs, assessed through the IPAQ (r = 0.439, *p* = 0.01). Age was positively associated with heart rate (r = 0.547, *p* = 0.013) and arterial stiffness, as measured by AI (r = 0.439, *p* = 0.05) and AIx@75 (r = 0.569, *p* = 0.009). Weight was positively correlated with SBP (r = 0.697, *p* = 0.001), while inversely associated with HR (r = −0.443, *p* < 0.05), AIx@75 (r = −0.444, *p* < 0.05), and CD15 levels (r = −0.457, *p* < 0.05). BMI was positively correlated with SBP (r = 0.630, *p* = 0.003). SBP was negatively correlated with HR (r = −0.430, *p* = 0.05) and positively associated with METs (r = 0.561, *p* = 0.01). HR was positively correlated with AIx@75 (r = 0.719, *p* < 0.001) and negatively associated with METs (r = −0.610, *p* < 0.01). RHI and the derived lnRHI were negatively associated with the adherence to MeD, measured by the 14-MEDAS (r = −0.483, *p* < 0.05 and r = −0.504, *p* < 0.05 respectively). Plasma levels of CD15 were negatively associated with plasma VCAM-1 concentration (r = −0.499, *p* < 0.05). VCAM-1 levels were positively correlated with METs (r = 0.598, *p* < 0.01) and MeD adherence (r = 0.468, *p* < 0.05).

### 3.3. Effect of Wild Blueberry and Placebo Treatment on Vascular Function

**Figure 4:**
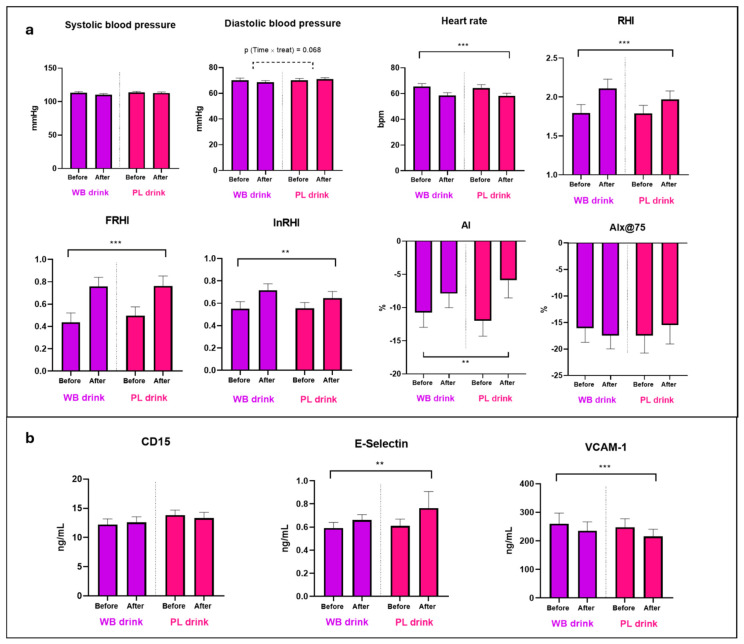
Peripheral vascular function parameters before and after (2 h) consumption of WB and PL drinks (**a**). Plasma levels of vascular endothelial markers before and after (2 h) consumption of WB and PL drinks (**b**). AIx = augmentation index; AIx@75 = augmentation index normalized to a heart rate of 75 bpm; CD15 = cluster of differentiation 15; DBP = diastolic blood pressure; FRHI = Framingham reactive hyperemia index; E-selectin = endothelial selectin; HR = heart rate; PL = placebo; RHI = reactive hyperemia index; SBP = systolic blood pressure; VCAM-1 = vascular cell adhesion protein-1; WB = Wild Blueberry. Solid brackets indicate a significant main effect of time, whereas dashed brackets indicate time × treatment interaction according to repeated-measures ANOVA. Asterisks denote the level of statistical significance (** *p* ≤ 0.01, *** *p* ≤ 0.001).

Figure 4a,b shows the effects of the interventions on vascular function measured 2 h post-consumption in the overall study population (*n* = 20). A significant main effect of time was observed, with increases in RHI, lnRHI, FRHI, AIx and E-selectin after both WB and PL treatment (*p* < 0.01). In addition, a significant reduction in HR and VCAM-1 was observed after the consumption of both beverages (*p* < 0.05). When comparing the WB and PL drink (time × treatment interaction), no significant differences were observed in vascular reactivity, arterial stiffness, systolic blood pressure, heart rate, or markers of vascular function, including CD15, E-selectin, and VCAM-1 (*p* > 0.05). However, a trend for a time × treatment interaction was observed for DBP, evidenced by a reduction following WB intake (*p* = 0.068).

Based on baseline RHI values, 12 participants were classified as having relatively lower endothelial function (RHI < 1.67). An exploratory subgroup analysis was therefore performed to investigate the response to the intervention in this subgroup (Table 3). Repeated measures ANOVA confirmed a significant effect of the time, but not of the treatment, for HR, RHI, lnRHI, FRHI, and VCAM-1 (*p* < 0.01). Specifically, HR and VCAM-1 decreased compared to baseline, while RHI, lnRHI, and FRHI increased.

## 4. Discussion

In this short-term study, we evaluated the effect of a WB drink versus a PL drink on vascular function and related biomarkers in healthy, young adults. Overall, the study did not demonstrate a treatment-specific effect of the WB drink on the primary endpoint (RHI) or on the other markers of vascular function compared with the placebo. Instead, a significant effect of time was observed following both interventions, with an increase in vascular reactivity (RHI, lnRHI and FRHI) and a reduction in VCAM-1, indicating that these temporary changes cannot be specifically attributed to WB consumption. Furthermore, the exploratory subgroup analysis based on vascular reactivity showed a similar time-dependent pattern among participants with lower baseline RHI values, although no treatment-specific effect was observed. Likewise, no significant treatment effects were detected for arterial stiffness, blood pressure, E-selectin and CD15.

Evidence from epidemiological and clinical studies hypothesized the contribution of PP-rich foods, including blueberries, in the modulation of vascular function, arterial stiffness and consequently blood pressure [40,41]. Several mechanisms have been proposed, including enhanced endothelial nitric oxide synthase activity, improved nitric oxide bioavailability, reduced oxidative stress, and modulation of inflammatory pathways [15]. The role of blueberries in the modulation of vascular function has been evaluated in several pre-clinical and clinical studies with conflicting findings. In our experimental conditions, we documented a lack of effect on RHI and related vascular markers following 2 h after the consumption of the WB drink in young healthy volunteers. The absence of the effect observed may be partly explained by the characteristics of the study population and/or by the methodology used to assess vascular function. Most evidence supporting the vascular benefits of blueberries has been reported in older adults or individuals at increased cardiovascular risk, including smokers and subjects with impaired vascular function [23,24,25,28,30,42,43]. In contrast, findings in young healthy individuals have been inconsistent [25], suggesting that any vascular benefits of blueberries may be more readily detected in the presence of endothelial dysfunction or reduced vascular homeostasis. However, even when the analysis was restricted to participants with lower baseline vascular function, the overall findings remained unchanged, with an apparent improvement in vascular function (RHI, lnRHI, FRHI) and reductions in VCAM-1 levels observed in both intervention groups regardless of the beverage consumed. This suggests that the changes were not specific to WB intake but may instead reflect postprandial metabolic responses to the beverages and their dietary components, particularly sugars, and/or interactions among these components [44,45,46,47,48].

Another possible explanation for the absence of an intervention effect relates to the assessment of vascular function. Previous blueberry intervention studies have found more positive findings when endothelial function was assessed via flow-mediated dilation (FMD) than via EndoPAT. Although both techniques are validated non-invasive measures of vascular function, they evaluate different vascular beds and reflect partially distinct physiological mechanisms, which may contribute to the discrepancies across studies [49,50]. In addition, the commonly used EndoPAT threshold (RHI < 1.67) was originally validated primarily in middle-aged individuals and populations at increased cardiovascular risk. Therefore, although this threshold was used in the present study to identify participants with relatively lower baseline endothelial function for exploratory subgroup analyses, its application to healthy young adults should be interpreted with caution.

In line with the RHI, arterial stiffness markers (AIx and AIx@75, AIx normalized to a heart rate of 75 bpm), we documented a lack of effect following the intervention. Arterial stiffness is a crucial marker of risk for hypertension and CVDs, which can be measured through pulse wave velocity (the gold standard method) or the surrogate marker AIx [51,52]. As with RHI, the effect of blueberries on arterial stiffness remains controversial in the literature. Several studies have found no significant effects on arterial stiffness, as measured by AIx or pulse wave velocity, following blueberry consumption, in line with our results [25,42,43,53]. However, following medium- to long-term blueberry consumption, previous studies have reported improvements in arterial stiffness markers [54,55,56]. The discrepancy with the present findings may be explained by differences in both the study population characteristics and the duration of the intervention [54,55], as changes in arterial stiffness are likely to require longer periods of exposure and are strongly influenced by age. Consistent with this, AIx@75 was positively associated with age in our study, supporting its role as a marker of vascular aging [51,52]. In addition, AIx@75 was inversely associated with physical activity and adherence to the MeD, in agreement with previous evidence showing that higher physical activity and greater adherence to the MeD are associated with lower arterial stiffness [51,57].

In agreement with our findings on vascular function and arterial stiffness, blood pressure was also unaffected by the intervention. The absence of an effect is in line with several previous studies conducted in young and healthy populations [16,25,53,58]. In contrast, reductions in blood pressure have been reported mainly following longer-term blueberry interventions or in populations at increased cardiovascular risk, including individuals with prehypertension or hypertension [55,59], metabolic syndrome [60], and older adults [15,61,62,63]. Similarly, the few acute studies showing beneficial effects in healthy young adults were performed under conditions of vascular stress, such as cigarette smoking [23,24]. Overall, these findings support the hypothesis that the blood pressure-lowering effects of blueberries may be more readily detectable in individuals with impaired vascular health or under conditions of increased vascular stress than in healthy young adults.

Functional vascular outcomes (RHI, AIx, and blood pressure) were assessed alongside circulating biomarkers of vascular activation and inflammation (VCAM-1, E-selectin and CD15) to provide a more comprehensive evaluation of the acute vascular response to this short-term intervention.

VCAM-1 and E-selectin are endothelial adhesion molecules induced by inflammatory stimuli that reflect endothelial activation and contribute to vascular inflammation by mediating distinct stages of leukocyte recruitment to the vascular wall. Specifically, E-selectin primarily mediates the initial rolling and recruitment of leukocytes, whereas VCAM-1 promotes their firm adhesion and subsequent transmigration into the vessel wall. Consistent with this, acute consumption of the WB drink did not influence circulating markers of endothelial activation in healthy young adults. Although VCAM-1 concentrations, but not E-selectin, decreased 2 h after the beverage intake, a comparable reduction was observed following both the WB and placebo drinks, suggesting a time-related effect rather than a specific response to WB consumption. These findings are in line with the limited available evidence, which has generally reported little or no effect of blueberry interventions on circulating endothelial adhesion molecules. Although reductions have occasionally been described following longer-term interventions or with other berry species, in subjects with increased oxidative stress, low-grade inflammation and cardiometabolic risk factors [53,60,64,65,66,67,68].

Similarly, WB consumption did not affect circulating CD15 expression compared with placebo. CD15 is involved in leukocyte adhesion and migration during inflammatory responses through interactions with endothelial selectins [69,70,71]. To the best of our knowledge, this is the first dietary intervention to investigate the acute effect of WB consumption on circulating CD15 levels. The lack of an acute effect may suggest that a single WB drink is insufficient to modify endothelial activation in healthy young adults, particularly in the absence of underlying endothelial dysfunction or heightened inflammatory and oxidative stress. Whether longer-term consumption or interventions in populations with greater inflammatory burden or cardiometabolic impairment may modulate CD15 remains to be established.

This study shows some strengths but also limitations. Regarding its strengths, the study was powered and included subjects of both sexes in equal numbers. In addition, the amount of WB in terms of freeze-dried (30 g) and beverage (300 mL) could be reasonably consumed as part of a balanced diet. On the other hand, the main limitations of the present study include: (1) the measurement of RHI at 2 h post WB ingestion only. Although most of the studies conducted by our group and others, evidenced an effect on vascular function in correspondence of plasma ACN peak (at 2 h), we cannot exclude that an RHI improvement occurred later (e.g., at 3–4 h post WB consumption); (2) the lack of analysis of circulating plasma PP/ACNs documenting that their maximum peak of absorption occurred at 2 h in correspondence of RHI measurement; (3) since an improvement in RHI has been documented also following the PL drink, the lack of measurement of glycaemia and related hormones such as insulin and glucagon can also present a limitation since the sugar concentrations contained in both drinks would be expected to transiently impair than improve vascular function; and (4) the lack of a direct assessment of plasma total antioxidant capacity (e.g., TEAC), since this would have complemented the measurement of circulating (poly)phenols and provided further insight(s) into the contribution of oxidative stress to the observed response.

## 5. Conclusions

In conclusion, evidence on the acute vascular effects of blueberries in healthy individuals remains limited. In this study, a portion of a WB drink rich in ACNs and other PPs did not significantly affect vascular function or related biomarkers in healthy young adults, possibly due to the age of the participants and their healthy physiological state. Well-designed, long-term intervention trials in subjects with CVD risk factors and/or vascular dysfunction are needed to clarify the role of WB in supporting endothelial health. Such studies should also adopt an integrated mechanistic approach combining vascular assessments with comprehensive metabolic and metabolomic profiling to better elucidate the biological pathways underlying the potential vascular effects of blueberries.

## Figures and Tables

**Table 1:** Nutritional and phenolic composition of PL and WB powder.

	PL Powder (30 g)	WB Powder (30 g)
Energy (kcal)	115	122
Total fat (g)	0	1.7
Protein (g)	0	0.8
Sugar (g)	20.2	21
Fructose (g)	10.6	10.8
Glucose (g)	9.6	10.4
Total dietary fibre (g)	6.7	5
Insoluble fibre (g)	5.4	4.3
Soluble fibre (g)	1.2	0.7
Total (poly)phenols (mg)	-	738
Anthocyanins (mg)	-	459
Chlorogenic acid (mg)	-	171

PL = placebo; WB = wild blueberry.

**Table 2:** Baseline subjects’ characteristics (*n* = 20).

Variables	Value	Min–Max
Age (y)	29.4 ± 6.3	23–43
BMI (kg/m^2^)	22.2 ± 2.4	18.7–26.3
SBP (mmHg)	115 ± 8.8	100–130
DBP (mmHg)	72 ± 5.6	62–84
HR (bpm)	67.5 ± 11.7	50–86
RHI	1.75 ± 0.43	1.13–2.49
lnRHI	0.53 ± 0.24	0.12–0.91
FRHI	0.45 ± 0.35	−0.13–1.07
AIx (%)	−12.4 ± 11.2	−34–10
AIx@75 (%)	−16.8 ± 14.8	−49–14
Glycemia (mg/dL)	89.6 ± 6.5	80–100
TC (mg/dL)	171.2 ± 37.6	112–243
LDL-C (mg/dL)	102 ± 30.2	58–158
HDL-C (mg/dL)	62.1 ± 8.8	42–80
TG (mg/dL)	81.1 ± 47.5	42–218
TC/HDL-C	2.8 ± 0.5	2.1–3.8
LDL-C/HDL-C	1.6 ± 0.5	1–2.5
TG/HDL-C	1.3 ± 0.8	0.6–3.1
AST (U/L)	21.2 ± 12.0	11–65
ALT (U/L)	19.5 ± 18.2	9.0–91.0
GGT (U/L)	15.7 ± 5.1	10.2–26.2
VCAM-1 (ng/mL)	268.7 ± 164.8	57–540
E-selectin (ng/mL)	0.6 ± 0.3	0.5–1.5
CD15 (ng/mL)	13.3 ± 4.4	6–22

Values are reported as mean ± standard deviation. AIx = augmentation index; AIx@75 = augmentation index normalized to a heart rate of 75 bpm; ALT = Alanine transaminase; AST = aspartate aminotransferase; BMI = body mass index; CD15 = cluster of differentiation 15; DBP = diastolic blood pressure; E-selectin = endothelial selectin; FRHI = Framingham reactive hyperemia index; GGT = Gamma glutamyl transferase; HDL-C = high-density lipoprotein cholesterol; HR = heart rate; LDL-C = low-density lipoprotein cholesterol; RHI = reactive hyperemia index; SBP = systolic blood pressure; TC = total cholesterol; TG = triglycerides; VCAM-1 = vascular cell adhesion protein-1.

**Table 3:** Effect of wild blueberry and placebo drink in subjects with vascular dysfunction (RHI ≤ 1.67) (*n* = 12).

Variables	Pre-WB	Post-WB	Pre-PL	Post-PL	Time	Treatment	Time × Treatment	η^2^p (Time × Treatment)
SBP (mmHg)	112 ± 2	108 ±1.7	112 ± 2.2	111 ± 1.8	0.126	0.471	0.274	0.054
DBP (mmHg)	70 ± 2.1	67 ± 1.7	71 ± 1.8	71 ± 1.2	0.108	0.356	0.200	0.074
HR (bpm)	68 ± 2.4	61 ± 2.6	68 ± 9.3	61 ± 2.3	<0.001 *	0.860	0.718	0.006
RHI	1.52 ± 0.1	2.07 ± 0.2	1.5 ± 0.04	1.9 ± 0.2	<0.001 *	0.511	0.461	0.025
lnRHI	0.39 ± 0.07	0.69 ± 0.09	0.4 ± 0.03	0.62 ± 0.07	<0.001 *	0.684	0.452	0.026
FRHI	0.27 ± 0.1	0.71 ± 0.12	0.29 ± 0.07	0.72 ± 0.11	<0.001 *	0.914	0.899	0.001
AIx (%)	−13 ± 3.2	−10.3 ± 3.1	−10 ± 2.9	−6.9 ± 3.3	0.094	0.447	0.921	<0.001
AIx@75 (%)	−16.4 ± 3.7	−17.2 ± 3.6	−14.1 ± 3.8	−15 ± 4.3	0.613	0.670	0.960	<0.001
CD15 (ng/mL)	11.5 ± 1.2	11 ± 1.2	13.1 ± 1.3	13.5 ± 1.1	0.915	0.171	0.626	0.011
E-selectin (ng/mL)	0.62 ± 0.08	0.67 ± 0.07	0.64 ± 0.1	0.82 ± 0.23	0.090	0.623	0.338	0.051
VCAM-1 (ng/mL)	270 ± 55.4	240 ± 44.4	259 ± 42.2	225 ± 35.4	0.007 *	0.829	0.848	0.002

Data are expressed as mean ± standard error of the mean. AIx = augmentation index; AIx@75 = augmentation index normalized to a heart rate of 75 bpm; CD15 = cluster of differentiation 15; DBP = diastolic blood pressure; FRHI = Framingham reactive hyperemia index; E-selectin = endothelial selectin; HR = heart rate; PL = placebo; RHI = reactive hyperemia index; SBP = systolic blood pressure; VCAM-1 = vascular cell adhesion protein-1; WB = wild blueberry. * Significant differences (*p* < 0.05).

## Data Availability

The data presented in this study are available upon request from the corresponding author.

## Clinical Trial Registration

This study was prospectively registered in the ISRCTN Registry (ISRCTN59129089) on 5 June 2014.

## Abbreviations

The following abbreviations are used in this manuscript:

AIx	Augmentation index
AIx@75	AIx adjusted to a heart rate of 75 bpm
BMI	Body mass index
CD 15	Cluster of differentiation 15 (or Lewis x)
DBP	Diastolic blood pressure
FRHI	Framingham RHI
FMD	Flow-mediated dilation
HR	Heart rate
PL	Placebo
RHI	Reactive hyperemia index
SBP	Systolic blood pressure
VCAM-1	Vascular cell adhesion molecule-1
WB	Wild blueberries
